# Correction: Jeong et al. *WBP5* Expression Influences Prognosis and Treatment Response in Head and Neck Squamous Cell Carcinoma. *Cancers* 2025, *17*, 587

**DOI:** 10.3390/cancers17152493

**Published:** 2025-07-29

**Authors:** Eun-jeong Jeong, Eunjeong Kim, Kwang-Yoon Jung, Seung-Kuk Baek, Yeon Soo Kim

**Affiliations:** 1 Department of Otorhinolaryngology–Head and Neck Surgery, Konyang University College of Medicine, Daejeon 35365, Republic of Korea; 602547@kyuh.ac.kr; 2 Department of Otorhinolaryngology–Head and Neck Surgery, Korea University College of Medicine, Seoul 02841, Republic of Korea; kyjung@korea.ac.kr (K.-Y.J.); mdbsk@korea.ac.kr (S.-K.B.); 3 BK21 FOUR KNU Creative BioResearch Group, Department of Biology, College of Natural Sciences, Kyungpook National University, Daegu 41566, Republic of Korea; eunjkim@knu.ac.kr

## Error in Figure

In the original publication [1], there was a mistake in Figure 6A as published. The gene label “WDR54” was incorrectly used instead of “WBP5”.

The correct figure is provided below:

**Figure 6:**
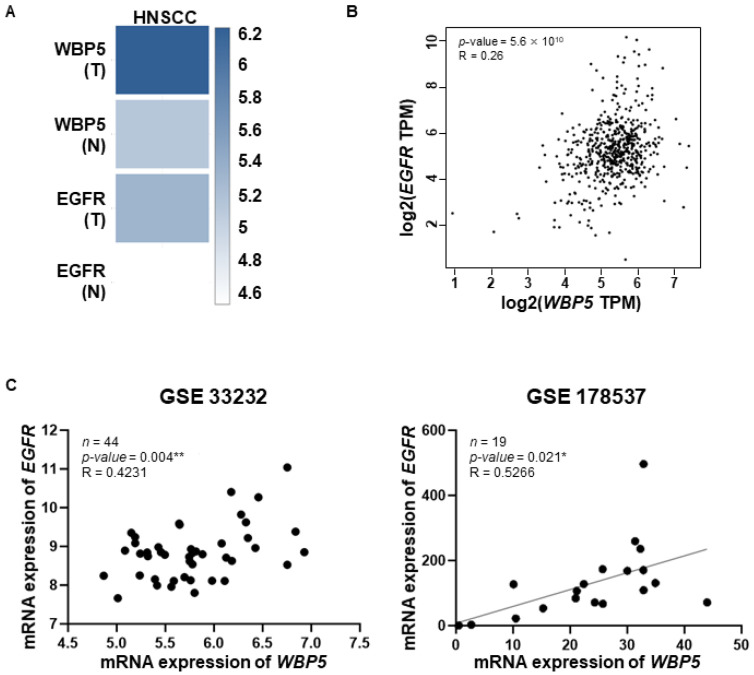
Correlation of *WBP5* and *EGFR* expression across different cancer types and datasets. (**A**) Heatmaps of *WBP5* and *EGFR* expression across HNSCC, with darker blue indicating higher expression levels. (**B**) Scatter plot showing the correlation between *WBP5* and *EGFR* expression (log2 TPM) across samples, with a positive correlation (R = 0.26, *p* = 5.6 × 10^10^). (**C**) Scatter plots showing the correlation between *WBP5* and *EGFR* expression in specific datasets (GSE33232 and GSE178537). Both datasets demonstrate a positive correlation between *WBP5* and *EGFR* expression, with GSE33232 (R = 0.4231, *p* = 0.004) and GSE178537 (R = 0.5266, *p* = 0.021) (* *p* < 0.05 and ** *p* < 0.01).

## Text Correction

There was an error in the original publication [1]. The protein name was incorrectly written as “WW domain C-binding protein 5 (*WBP5*)”, instead of “WW domain protein 5 (*WBP5*)” in the Introduction, paragraph 2.

The correct text is provided below:

“WW domain protein 5 (*WBP5*), alternatively referred to as Transcriptional Elongation Factor A-like 9 (*TCEAL9*), engages with diverse signaling pathways and associated with WW domains [3]”.

A correction has been made to Materials and Methods, 4.8. Cell Culturing and Viability Assessment, where “WDR54-knockdown FaDu cells” was incorrectly written instead of “WBP5-knockdown FaDu cells”.

The correct text is provided below:

“Assays were conducted using WBP5-knockdown FaDu cells. For the cell viability experiments, 3 × 10^3^ cells/well were seeded into 96-well plates, while 1 × 10^3^ cells/well were plated for proliferation analysis.”

The authors apologize for any inconvenience caused and state that the scientific conclusions are unaffected. This correction was approved by the Academic Editor. The original publication has also been updated.

## References

[1] Jeong E.-j., Kim E., Jung K.-Y., Baek S.-K., Kim Y.S. (2025). *WBP5* Expression Influences Prognosis and Treatment Response in Head and Neck Squamous Cell Carcinoma. Cancers.

