# Supplementary figures and images for: WBP5 Expression Influences Prognosis and Treatment Response in Head and Neck Squamous Cell Carcinoma
Source: Cancers (Basel). 2025 Feb 8;17(4):587. doi: 10.3390/cancers17040587 (PMC11852431; doi:10.3390/cancers17040587)

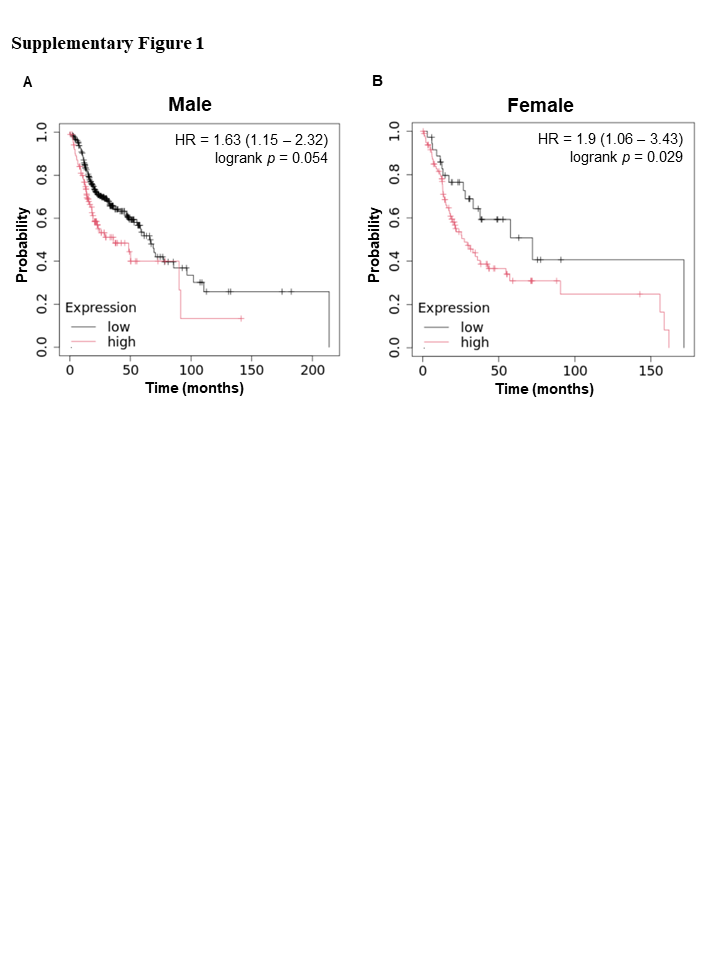

Supplement: Supplementary file 1 [file cancers-17-00587-s001.zip › sup-fig 1.TIF]

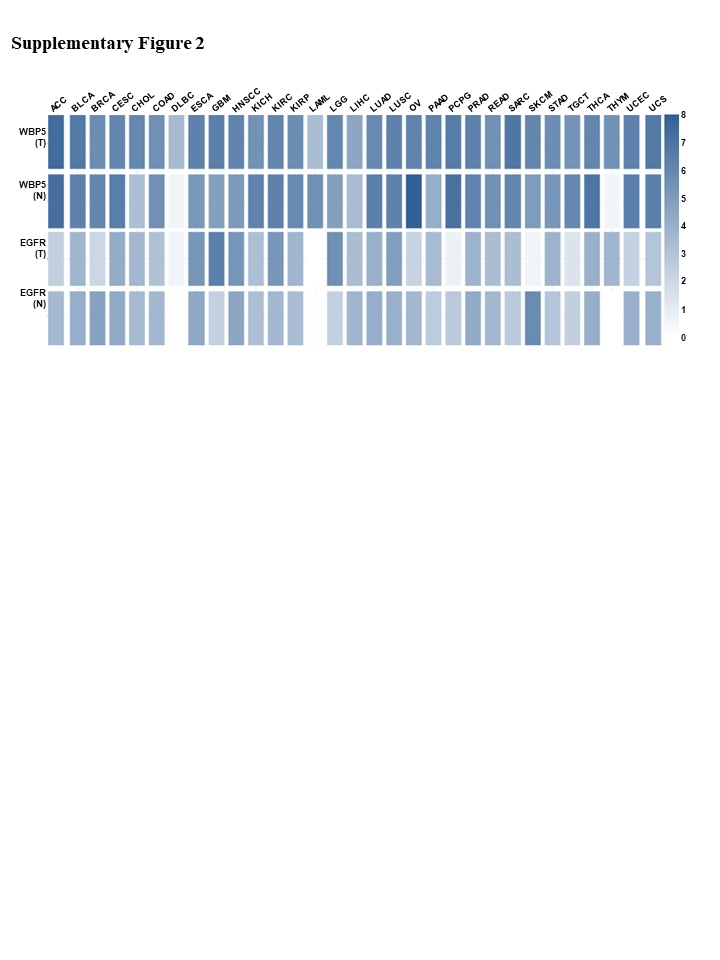

Supplement: Supplementary file 1 [file cancers-17-00587-s001.zip › sup-fig 2.TIF]
